# Two golden anniversaries in the biological sciences

**DOI:** 10.1128/msphere.00240-25

**Published:** 2025-05-14

**Authors:** Michael J. Imperiale, Arturo Casadevall

**Affiliations:** 1 Department of Microbiology and Immunology, University of Michigan 1259, Ann Arbor, Michigan, USA; 2 Department of Molecular Microbiology and Immunology, Johns Hopkins University Bloomberg School of Public Health 25802, Baltimore, Maryland, USA

**Keywords:** bioweapons, Asilomar, BWC

## EDITORIAL

Anniversaries have a way of focusing our attention on past events because they provide the opportunity to assess their consequences with the hindsight of history. The pace of discovery in biological science has been so breathtaking that it is difficult to choose discrete events as standing out since they are often interconnected, and their implications continue to be incompletely understood or appreciated. However, 2025 is the golden anniversary of two important events at the intersection of science and society with profound consequences that reverberate in our time. In February and March of this year, we marked the 50th anniversaries of the Asilomar meeting about recombinant DNA (24–27 February 1975) and the date the Biological Weapons Convention (BWC) came into effect (26 March 1975). The Asilomar meeting was organized by individual scientists, while the BWC was an international treaty organized by nation states. Both had in common the restriction of certain activities in biological research with the goal of increasing safety and reducing human suffering. However, they differed in that Asilomar was focused on concerns about possible harm from unknowns in future research, while the BWC sought to prevent the use of known and future agents and toxins in biological warfare.

“Asilomar,” which in the parlance of the scientific community has come to mean the conference rather than the beautiful state park in California where the conference was held, was a seminal event in the life sciences. For the first time in history, biologists and others came together and decided to desist from doing certain experiments. The conference was catalyzed by the advent of molecular biology, which provided the ability to splice together pieces of DNA from different organisms, creating recombinant DNA (rDNA) molecules that did not exist in nature. Some scientists worried about the potential risks associated with this capability. The community enacted a moratorium and convened a conference to discuss the issues. While there has been criticism over the years about whether all the appropriate stakeholders were at the table, the result of the conference was the consensus that the field needed to proceed with caution due to the uncertainties surrounding what might happen if a researcher were exposed to rDNA or if such molecules escaped the laboratory. This led to the development in the United States of the NIH Guidelines for Recombinant DNA Research, affectionately known as “the NIH Guidelines,” which spelled out the types of physical containment and experimental protocols that should be implemented, based on what was known about the potential risks at that time. Compliance with the Guidelines was, and still is, required of all institutions receiving NIH funding, and even private companies that were not recipients of NIH dollars have complied voluntarily. Over the years, as the field gained experience and learned more about the risks, the Guidelines have been updated (relaxed in some areas and augmented in others) and now include work with synthetic DNA molecules. This was a remarkable success story that eventually produced an enormous bounty of products useful to humanity, such as human insulin, vaccines, pest-resistant crops, and many, many more.

The Biological Weapons Convention (BWC) is the treaty that prohibits the development, production, and stockpiling of biological and toxin weapons. It is the only international agreement since World War II that called for the elimination of an entire class of weapons. Among nations, 188 states are parties to the treaty, with another four that have signed it but not ratified it. Only five states have neither signed nor ratified. The BWC was a remarkable achievement in diplomacy. While there are no provisions in place for verification, in part due to the difficulties identifying biological weapons programs (as opposed to nuclear weapons programs, for example), the fact is that there have been no documented cases of one state attacking another with a biological agent in these 50 years. The signatories continue to meet on a regular basis to discuss advances in technology that might affect the ability to design and produce biological weapons.

These two activities are especially relevant to us microbial scientists, as it is infectious agents that pose exactly the types of risks that led to Asilomar and the BWC. Both Asilomar and the BWC established norms for limiting risky biological research that echo in the current debates about oversight of research into pathogens with pandemic potential, gain-of-function experiments, and synthetic biology. They both provided mechanisms for learning and reconsidering risk. The concerns expressed at Asilomar about the creation of Frankenstein-type microbes appear to have been greatly overstated, and with time, the Guidelines were loosened to facilitate the molecular biology revolution that has revolutionized medicine and more distant fields such as criminology (DNA evidence). Nevertheless, the molecular biology revolution continues to pose new moral and ethical challenges since it has also facilitated the possibility of designer babies, human cloning, and the resurrection of long-extinct species. Other new challenges were discussed on the 50th anniversary of Asilomar at a conference entitled The Spirit of Asilomar and the Future of Biotechnology. The BWC created limits that have kept us safe from biological warfare, although those concerns and fears never really subsided, especially in the wake of several instances of domestic terrorism with biological agents and occasional rogue groups that have invested in biological weapons. Hence, in considering the golden anniversaries of Asilomar and the BWC, we can take solace in the idea that humanity can act responsibly, which provides a successful precedent as we face new challenges in biological research. As we mark these anniversaries, let’s pause and remind ourselves of our responsibilities as scientists to adhere to the principles set out 50 years ago.

